# Acute methamphetamine and alcohol usage alters gaze behaviour during driving: A randomised, double-blind, placebo-controlled study

**DOI:** 10.1177/02698811241261024

**Published:** 2024-07-28

**Authors:** Amie C Hayley, Brook Shiferaw, Blair Aitken, Joanna Rositano, Luke A Downey

**Affiliations:** 1 Centre for Human Psychopharmacology, Swinburne University of Technology, Hawthorn, VIC, Australia; 2 Institute for Breathing and Sleep, Austin Hospital, Melbourne, VIC, Australia; 3 International Council for Alcohol, Drugs and Traffic Safety; 4 Forensic Science SA, Adelaide, South Australia, Australia

**Keywords:** Methamphetamine, alcohol, driving, randomised controlled trial, gaze, eye movement

## Abstract

**Background:**

Methamphetamine is frequently co-consumed with alcohol, yet combined effects on visually guided behaviours have not been experimentally assessed. This study examined whether methamphetamine and alcohol-induced changes in gaze behaviour can be accurately detected and indexed during a simulated driving task to establish characteristic patterns relevant to traffic safety.

**Methods:**

In a randomised, placebo-controlled, cross-over study design, the effects of acute oral methamphetamine (0.42 mg/kg) were assessed with and without low doses of alcohol (target 0.04% blood alcohol content) on gaze behaviour during driving. Twenty healthy adults (mean age 29.5 years (SD ± 4.9), 40% female) completed four, 1-h simulated drives with simultaneous eye monitoring using the SensoMotoric Instruments cap-mounted eye tracker over a 4-week experimental paradigm. Gaze entropy measures were used to quantify visual scanning efficiency, expressed as gaze transition entropy and stationary gaze entropy. Fixations, recorded as duration (milliseconds, ms) and rate (count) per minute, were examined in 10-min bins over the duration of the drive. Driving performance was assessed by the standard deviation of lateral position, standard deviation of speed and steering variability.

**Results:**

Methamphetamine increased the rate and duration of fixations and produced a less dispersed but more disorganised pattern of gaze during highway driving while preserving performance. Alcohol alone impaired both oculomotor control and driving performance, even when consumed at levels well below the legal limit stipulated in many international jurisdictions.

**Conclusions:**

Methamphetamine-affected drivers display inefficient exploration in a limited visual range during driving. Eye-tracking metrics thus show potential for indexing intoxication due to psychoactive substance usage.

## Introduction

Amphetamine-type substances rank second only to alcohol in terms of global traffic-related morbidity and mortality and are rapidly surpassing other drugs as the most prevalent illicit substance detected among apprehended drivers (DiRago et al., 2021). Methamphetamine is one of the most prevalent amphetamine congeners consumed across North America, Africa, Asia and Oceania (Huizer et al., 2021) and it is associated with a considerable proportion of traffic-related harm in these regions. Methamphetamine-affected drivers are 19 times more likely to be culpable for a traffic crash resulting in serious injury compared to sober drivers (Drummer et al., 2020), and more than 1 in 10 people who use methamphetamine recreationally acknowledge having driven under the influence of the substance in the past year (Hayley et al., 2019). Despite this, the mechanism by which methamphetamine intoxication translates to increased traffic risk remains unclear. Unlike alcohol and other drugs, there is no clear delineation between drug concentration and accident risk, nor is there yet a useful model for approximating risk across different user groups. People who use methamphetamine have reported engaging in risky driving behaviours, such as speeding. Yet, participation in these behaviours is not attributable to negative emotional attributes such as aggression (Hayley et al., 2019), or changes to impulsivity due to acute intoxication (Arkell et al., 2022a). Complicating this further, acute stimulant use typically *improves*, rather than *impairs*, skills that are ostensibly related to driving when assessed in a laboratory (Dolder et al., 2018). Evidence of an inverted U-shaped concentration–effect relationship suggests that while low to moderate doses of amphetamine may have selected beneficial effects on neurocognitive functions, performance at higher doses is compromised, resulting in cognitive inflexibility and stereotyped rigid patterns of behaviour (Narayan et al., 2021). Secondary effects of sleep deprivation are also shown to dominate any acute beneficial effects produced by amphetamine, further compounding performance-based impairment in safety-relevant tasks like driving (Ramaekers et al., 2012). Importantly, any limited beneficial effects on performance relevant to driving appear to be entirely extinguished by co-consumption of pharmacologically opposing substances like alcohol (Narayan et al., 2021).

Driving is a safety-critical task that is principally reliant on sustained vigilance, visual acuity and the continuous seamless integration of attentional engagement with motor coordination. It demands a high cognitive load to facilitate constant scanning, selective prioritisation and efficient processing of a dynamically changing visual environment. These fundamental neurocognitive processes are susceptible to both internal (e.g. the influence of consumed psychoactive substances) and external factors (e.g. task difficulty or traffic complexity; Shiferaw et al., 2019a). Methamphetamine consumption induces acute dopaminergic alterations to contiguous cortical and subcortical regions responsible for voluntary movement (Volkow et al., 2001), behavioural inhibition (Heal et al., 2013) and ocular function (Arkell et al., 2022b; Pierrot-Deseilligny et al., 2005); systems that play a vital role in the selection and prioritisation of visual information during driving (Hayley et al., 2020). Acute intoxication produces a significant mismatch in these systems, leading to a narrowing of exploratory gaze patterns in each field of view. This alteration affects the frequency, spatial distribution and scanning pattern used to sample available visual information and process it cognitively, leading to what is termed ‘amphetamine-induced tunnel vision’ (Hayley et al., 2021). Such alterations to oculomotor control are likely to reduce the capacity to gather sufficient visual information necessary for accurate and efficient responses, compromising the ability to safely control a vehicle. Alterations in visual scanning behaviour have previously been demonstrated under acute alcohol conditions in a simulator environment (Shiferaw et al., 2019b), but this is yet to be replicated with centrally stimulating agents like methamphetamine, either when consumed alone or when combined with alcohol. Eye-tracking provides a non-invasive means to examine alterations in operator state, and is an effective tool for mapping the ascending and descending phases of alcohol intoxication (Shiferaw et al., 2019b), sleep deprivation (Shiferaw et al., 2018) and the degree to which they mediate and reasonably predict impairment in driving performance (Shiferaw et al., 2019c). Despite this, no studies have yet explicitly characterised the magnitude of methamphetamine and alcohol-related effects on ocular function during driving under an acute dosing paradigm.

Psychoactive substances directly alter eye movements, and the significance of these changes is magnified during dynamic, safety-critical tasks such as driving. Given the persistent and unresolved challenges associated with methamphetamine use in global road safety outcomes, this study aimed to investigate, for the first time, the effects of methamphetamine, both individually and in combination with alcohol, on visual scanning efficiency and simulated driving performance. Furthermore, this work sought to determine the utility of non-invasive eye-tracking methods in detecting and monitoring altered driver states resulting from methamphetamine and alcohol intoxication.

## Materials and methods

### Participants

**Figure 1.**
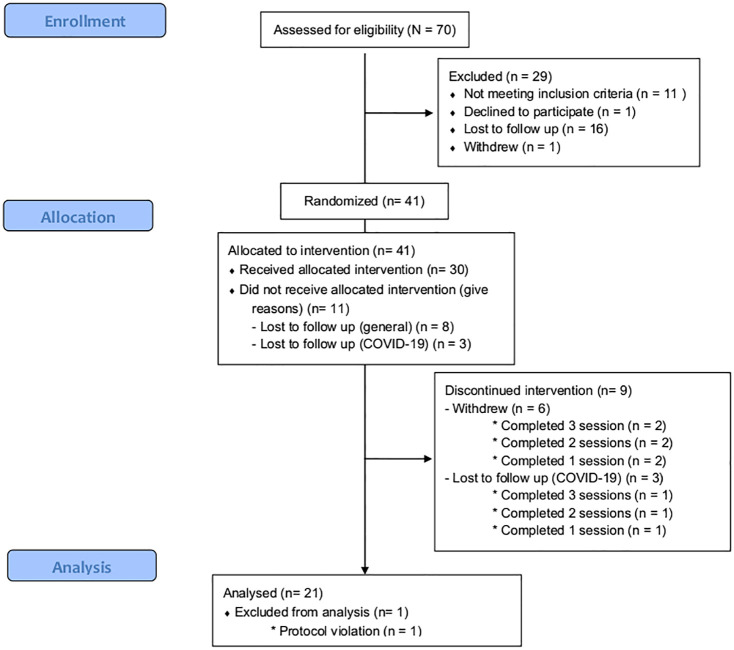
Adapted CONSORT diagram.

A total of 41 participants were recruited from the community between September 2018 and March 2022 via physical flyers and online advertisement campaigns. Due to significant COVID-19-related interruptions, analyses were performed on 21 participants who completed all study sessions and for those who had complete driving data (Consolidated Standards of Reporting Trials; CONSORT diagram, see Figure 1). For inclusion, participants had to be between 21 and 40 years old, self-report previous recreational (less than 2 times/month) use of amphetamine-type substances (methamphetamine, amphetamine, methylenedioxymethamphetamine, dexamphetamine, methylphenidate) and weigh less than 100 kg. All participants held a current valid (full) driver’s licence (Australian or international equivalent) for a minimum of 3 years and drove more than 4000 km per year. Exclusion criteria were current use of any medication for the treatment of a medical condition (except oral contraceptives in women or treatment for benign conditions, as determined by research physician), a current or past diagnosis of a physical, gastrointestinal, neurological or psychiatric condition, personal history of head-injuries or loss of consciousness (determined via clinical interview), blood pressure above 160/90 mmHg, and for women, a positive pregnancy test at the start of each study visit. A telephone interview was first conducted with individuals who expressed interest in participating in the study. If all inclusion criteria were met, they were invited for a clinical interview. A medical screening was first performed by a research nurse and eligibility was confirmed by a physician. Participants provided written informed consent prior to testing procedures and were reimbursed. The study was prospectively registered on the Australian New Zealand Clinical Trials Registry (ACTRN: 12618000629235) and was approved by the Swinburne University of Technology Human Research Ethics Committee (HREC: 20210770-8822).

### Biological sampling

Venous whole blood (5 mL) was drawn using a vacuum tube from a cannula placed in the cubital fossa vein or via venepuncture technique of the non-dominant arm at 30 min, 1 h, and finally at ~3 h post-treatment. Where cannulation was used, the line was first flushed with 10 mL 0.9% NaCl to avoid specimen contamination. Discard blood volume (5 mL) was drawn before each collection to prevent contamination with 0.9% NaCl solution. Once the sample was taken, the line was again flushed with 5 mL of 0.9% NaCl. Whole blood samples were immediately stored at −80°C.

### Treatments

#### Methamphetamine

The active treatment was 0.42 mg/kg methamphetamine (purity 99.8 ± 1.3%). Weighted doses were rounded to the nearest whole number (e.g. weight of 74 kg × 0.42 mg = 31.08 mg, provided 30 mg). An upper weight limit of 100 kg was applied to ensure that concentrations of methamphetamine and alcohol did not exceed 40 mg (rounded methamphetamine dose). Australian Measurement Institute provided raw product (batch numbers 14-D-12 and 19-D-01) and compounded into 5, 10 and 20 mg size #00 opaque capsules with microcrystalline cellulose filler. Identical matched placebo capsules comprised microcrystalline cellulose only.

#### Alcohol

The beverage was vodka (40% alcohol, Absolut brand) measured according to each participant’s body weight for a target blood alcohol content (BAC) of 0.04% (0.38 g alcohol/kg body weight) mixed with 300 mL orange juice. The unit of measure used in this study is grams of alcohol per 210 L of breath (g/210 L) which is equivalent to 0.04% BAC at g/100 mL of blood, measured to a precision of 0.001%. The maximum weight of participants enrolled was capped at 100 kg (~90.25 mL of Vodka per 300 mL beverage) to (a) mask the presence of Vodka and (b) not exceed three standard drinks in any one beverage. The placebo condition consisted of orange juice with ~3 mL of vodka floated on top for masking. Breathalyser readings were taken using a Lion Alcolmeter SD400PA that was calibrated quarterly by the Road Policing Division, Victoria Police.

### Measures

#### Eye tracking and gaze analysis

Visual scanning behaviour was assessed through fixation rate (number), fixation duration, gaze transition entropy (GTE) and stationary gaze entropy (SGE) using the SensoMotoric Instruments (SMI, Teltow, Germany) cap-mounted binocular (left side) combined pupil and corneal reflection tracker with 50 Hz sampling rate. The SMI equipment is a video-based mobile eye tracking system hardware with eye and scene cameras, connected to a laptop with iView (recording) and BeGaze (analysis) software. Prior to the driving task, a five-point horizontal/vertical calibration process was undertaken. Ocular events were filtered using the SMI BeGaze analysis software’s fixed dispersion-based event detection algorithm, set at a maximum dispersion of 100 px and a minimum duration of 80 ms. For entropy calculations, the valid area of interest (850 × 650 px) was divided into 884 state spaces of 25 × 25 px, resulting in a maximum entropy of log_2_(884) = 9.79 (SensoMotoric Instruments, 2011). The following parameters were obtained for each minute of driving:

1. SGE: SGE applies Shannon’s entropy calculation to the probability distribution of fixation locations within the valid area of interest to calculate the level of uncertainty in the spatial distribution of a sequence of fixations. Higher values indicate a wider distribution of fixations, reflecting a greater dispersion of gaze. Lower values indicate a more narrowed gaze distribution (Krejtz et al., 2014).
2. GTE: GTE applies the conditional entropy equation to Markov chain matrices of fixation transitions to calculate an overall measure of the predictability of visual scanning patterns. Higher values indicate a less structured, or more random, pattern of scanning behaviour (Krejtz et al., 2014). Entropy values (measured in bits) were normalised by dividing by the maximum possible entropy, with the normalised 0 values reflecting minimum entropy, and 1 reflecting maximum entropy (Shiferaw et al., 2018).
3. Fixation duration: It is measured in milliseconds and reflects the average (mean) time per minute that a driver held their gaze at a specific location. Longer fixation duration has been shown to reflect greater visual and mental processing demands during driving (Marquart et al., 2015).
4. Fixation rate: It is measured as rate (count) per minute and reflects the number of times a driver gazed at a specific location on the screen.

#### Driving simulator

Driving performance was assessed using the UC win/Road driving simulator (Software version 11.2; Forum 8, Tokyo, Japan). Simulator hardware comprised a stationary vehicle base (driver seat, functional dashboard and centre console) with control (steering, brake, speed, gear, indicator) and surround audio-visual display. The visual task display was presented on three separate 144 × 81 cm (weight by width) monitors with 1920 × 1080 px resolution at 60 Hz refresh rate. The adjustable driver seat was positioned approximately 180 cm from the centre computer monitor. Participants were instructed to maintain a central position within the left-hand lane of the dual carriageway (in accordance with Australian driving regulations), keep their speed at 100 km/h on the speedometer and overtake slower-moving vehicles as they appear on the roadway. A 60-min highway drive was chosen due to demonstrable fatigue effects within this time frame (Vakulin et al., 2007).

Primary driving outcomes were standard deviation of lateral position (SDLP), average speed (km/h), standard deviation of speed (SDS) and steering variability (SV). SDLP is considered the most reliable and sensitive measure for detecting alcohol-related performance impairment in simulated driving studies (Irwin et al., 2017). Deviation of the vehicle from the centre of the lane is recorded in centimetres, with negative and positive numbers representing left and right deviations respectively. The standard deviation of these values across the driving duration provides the SDLP variable, which reflects the capacity to maintain lane position (Verster and Roth, 2012b). Data points related to intentional lane deviation (such as lane changes) were removed prior to SDLP calculation. SV was used to assess the ability to maintain lane position and steering wheel control. Scores ranged from −1 (extreme left) to 1 (extreme right), with 0 being centred position of the steering wheel (Hayley et al., 2018). Average speed (km/h) and variability were assessed throughout the entire drive. The first and last 5 min of the driving task were omitted from the analysis to account for variability in performance upon commencing the driving task (i.e. where participants are approaching speed; Hayley et al., 2018; Manning et al., 2023).

### Perceived driving performance

The Perceived Driving Quality Scale (Verster and Roth, 2012a) was used to assess subjective driving performance following the driving task. Participants rated their driving performance on a scale from 0 (‘I drove exceptionally poorly’) to 100 (‘I drove exceptionally well’), around a midpoint of 50 indicating ‘I drove normally’.

### Bioassay

Methamphetamine analysis was carried out by Forensic Science SA, per the method described by Rositano et al. (2016) with some modifications. Methamphetamine quantitation was performed by spiking drug-free whole blood (Australian Red Cross) with increasing known amounts of methamphetamine. The calibration range was 5–1000 ng/mL, using D5-methamphetamine (Cerilliant) as the internal standard, and spiked to give a resulting concentration of 50 ng/mL. The method was linear with the limit of quantitation set at 5 ng/mL.

In brief, duplicate whole blood samples (150 µL) were diluted with aqueous ammonia (8%, 390 µL) and internal standard added (30 µL, 250 ng/mL). The samples were then vortexed and centrifuged (3000 rpm, 10 min). The samples were placed onto a Perkin Elmer Janus Liquid Handling Platform and 350 µL of supernatant was loaded onto a Supported Liquid Extractio (SLE) sorbent (Isolute^®^ SLE+400 96-well plates; Biotage Isolate®, Uppsala, Sweden). After 5 min absorption time, the samples were eluted into a 96-well collection plate containing glass inserts, with methyl t-butyl ether (2 × 850 µL; Sigma-Aldrich, Macquarie Park, NSW, Australia), evaporated and reconstituted in methanol (LiChrosolve^®^, 80 µL). The collection plates were capped and centrifuged (2000 rpm, 5 min) then transferred to a Shimadzu Nexera LC system fitted with a Restek Ultrabiphenyl (3 µm × 50 mm × 2.1 mm) column and Phenomenex 2.1 mm PFP guard cartridge. The mobile phase was a gradient of acetonitrile (Optima LC/MS grade) and 0.1% formic acid (Optima) over 6 min with a flow rate of 0.5 mL/min. Liquid Chromatography/Mass Spectrometry (LC/MS) analysis was performed on a SCIEX QTrap 5500 mass spectrometer in positive ion mode with scheduled multiple reaction monitoring of three transitions: m/z 150/119, m/z 150/91, m/z 150/65 with m/z 150/119 used for quantitation and all three transitions used for drug identification.

Method validation gave a coefficient of determination (*r*^2^) for the calibration curve of greater than 0.999, the curve weighting was 1/× and was not forced through zero. Drug-free blood sample replicates (*n* = 96) spiked at concentrations of 5, 12.5, 117.5 and 500 ng/L on 3 separate days giving intra-day and inter-day accuracy from 94% to 104% and relative standard deviation of less than 9%. Matrix effects for nine different ante-mortem blood samples were between 0.80 and 0.96.

A fully validated method of alcohol analysis for legal blood alcohol concentration for forensic samples was used. The method’s lower and upper limit of quantitation is 0.01 and 0.50 g/dL. Essentially 50 µL blood samples, diluted 1:9 with an acidified aqueous n-propanol internal standard solution (0.0125 g/dL, 0.07 N H_2_SO_4_) are precipitated with aqueous sodium tungstate (50 µL, 88 mg/mL) in glass Gas Chromatography (GC) vials. The samples are vortexed, centrifuged (4000 rpm, 10 min) and analysed.

Analysis was performed on an Agilent 8890 gas chromatograph with dual flame ionisation detectors fitted with front and rear columns 30 m × 0.53 mm, 2 µm DB-ALC2 and 30 m × 0.53 mm, 1 µm HP-INNOWAX respectively with the rear column only used for quantitation. High-purity nitrogen was used as the carrier gas. Quantitation was achieved by analysis of certified aqueous ethanol standard solutions (Lipomed, Switzerland) having calibration range of 0.02–0.20 g/dL. A certified aqueous ethanol standard solution of 0.05 g/dL was used as a quality control (Cerilliant, Round Rock, Texas, United States). All samples positive for alcohol were re-analysed on a separate day and the average of the duplicates was reported.

Method validation gave a coefficient of determination (*r*^2^) for the calibration curve greater than 0.9999 (*n* = 6). Validation was carried out with replicate analysis of spiked blood samples having concentrations ranging between 0.01 and 0.50 g/dL (*n* = 360). Overall inter-day and intra-day accuracy was 100% with an overall relative standard deviation of 1.7%.

### Procedure

This trial used a double-blind, placebo-controlled, randomised, four-way within-subjects design with a minimum 1-week wash-out period between study visits. Each participant received 0.42 mg/kg methamphetamine and vodka drink (with matched placebo capsule or drink) in a randomised order. Study visits occurred at the same time each day to control for any circadian variation in performance. The order of administration and treatment combination was determined via computer-generated randomisation software (Urbaniak and Plous, 2013). Researchers were blinded and not involved in generating the randomisation schedule nor allocating participants to the different drug conditions. Allocation concealment was maintained by uniformly providing the same number of capsules or drink blinding, thereby preventing biases related to treatment effects.

Upon arrival at the testing site, participants first underwent a breathalyser analysis to confirm the absence of alcohol use, oral fluid screening to confirm the absence of drug use (Drugwipe 6s screen; cannabis, opiates, cocaine, amphetamines/methamphetamines/ecstasy and benzodiazepines) and medication/lifestyle history was reviewed to determine ongoing eligibility. Alcohol and caffeine were prohibited for 12 h, and participants were required to consume a standard breakfast, no later than 2 h prior to each visit. Female participants provided a urine sample to confirm the absence of pregnancy. Participants were then provided with a combination of capsules and a drink according to the scheduled treatment allocation. Performance measures (driving task) were completed 1.5–2.5 h post-treatment ingestion when peak plasma levels would be expected to be reached. Upon completing the simulated drive, participants rated their perceived driving quality. Participants were prohibited from driving to/from the testing visit (and for 24 h post-testing) and were provided a taxi voucher upon site departure.

### Statistical analysis

Raw data for ocular parameters (SMI) were processed using R (RStudio 1.1.463, Inc., Boston, MA, USA). Driving and ocular data were analysed in 5-min time bins and as a function of the whole drive. SGE and GTE were determined with previously applied parameters (see Shiferaw et al., 2019a), calculated for each trial, normalised and then averaged across each participant. Linear fixed effects models with restricted maximum likelihood estimation were used to examine the objective driving performance outcomes per treatment and across the driving task, segmented into 10-min bins. Using the likelihood ratio statistic to determine the best-fitting variance, compound symmetry was considered best fit. Time and condition were entered into the model as the repeated factors, and separate models were built to examine driving performance indicators (SDLP, SDS and SV). Univariate analysis of variance was used to examine differences in treatment between subjective driving performance outcomes. Where a main effect was observed, post hoc paired *t*-tests with Bonferroni correction for multiple comparisons were conducted to contrast each time point from placebo and other active dosing conditions. Finally, linear regression models were used to assess associations between biological matrices and (a) performance and (b) subjective outcomes. Separate models were also developed to investigate associations between gaze metrics and driving performance. All statistical analyses were conducted using SPSS 28.0.1.1 (IBM Corp, 2013).

## Results

### Demographics

Results from *n* = 1 participant were found to have violated protocol after unblinding. As such, analysis was conducted on the remaining 20 individuals (12 males). Driving data for *n* = 1 time bin (60 min) were missing for one participant, representing a usable data loss of 0.2%.

Demographic and driving characteristics are shown in Table 1. The mean age of participants was 29.5 years (SD ± 4.9), the mean body mass index was 25.4 kg/m^2^ (range 20.2–33.9 kg/m^2^) and the sample predominantly comprised those who self-reported their ethnicity as Caucasian (95%). The majority reported themselves as experienced (90%) or moderately experienced drivers (10%), holding their full driver’s licence for an average of ~10 years and driving an average of 183 km/week. All participants self-reported having used amphetamine-type stimulants, cannabis and alcohol at least once in their lifetime. Additionally, a large majority reported having consumed cocaine (85%). The average (weighted) methamphetamine dose provided during experimental sessions was 31.5 mg, while the average (weighted) amount of alcohol was 76.74 g.

**Table 1.** Demographic and driving characteristics for the whole sample (*N* = 20).

Demographic parameters	Mean (±SD); *n* (%)
Sex, *n* (%)	
Female	8 (40)
Weight (kg), (±SD)	
BMI (kg/m^2^)	25.4 (3.8)
Age (years), (±SD)	29.5 (5.0)
Hours driven per week	183.8 (77.8)
Hours driven per year (×1000)	65.5 (143.5)
Handedness, *n* (%)	
Right-handed	18 (19)
Race/Ethnicity, *n* (%)	
Caucasian	19 (95)
Other/mixed	1 (5)
Employment status, *n* (%)	
Part-time/casual	12 (60)
Full-time employment	7 (35)
Unemployed	1 (5)

SD: standard deviation; BMI: body mass index.

#### Bioassay

Whole blood concentrations of methamphetamine (ng/mL) and alcohol (g/dL (%w/v)) per condition and timepoint are presented in Table 2. Methamphetamine concentrations for the methamphetamine and methamphetamine and alcohol condition(s), and alcohol levels for the alcohol and methamphetamine and alcohol condition(s) were comparable at each collection timepoint (all *p* > 0.05). Peak blood alcohol concentration was 0.025 g/dL for the alcohol condition and 0.032 g/dL for the methamphetamine and alcohol condition, both reported at Time 2. Whole blood concentrations of methamphetamine were comparable between the methamphetamine and methamphetamine and alcohol condition(s), respectively, at 3.42 ng/mL (range 00.00–36.0 ng/mL, SD ± 10.85) and 0.40 ng/mL (range 0.0–6.0 ng/mL, SD ± 1.81) at Time 1, 31.69 ng/mL (range 0.00–78.00 ng/mL, SD ± 30.90) and 35.53 ng/mL (range 0.00–73.00 ng/mL, SD ± 24.82) at Time 2 and 92.20 ng/mL (range 53.00–120.00 ng/mL, SD ± 18.50) and 93.33 ng/mL (range 67.00–110.00 ng/mL, SD ± 15.49) at Time 3.

**Table 2.** Whole blood concentrations of methamphetamine (ng/mL) and alcohol (g/dL (%w/v)) per condition and timepoint presented as mean (±SD).

Time since dosing (min)	Whole blood alcohol concentration (g/dL (%w/v))		Whole blood methamphetamine concentration (ng/mL)	
	Alcohol	Alcohol + methamphetamine	Methamphetamine	Alcohol + methamphetamine
30	0.02 (0.01)	0.03 (0.01)	3.42 (9.91)	0.40 (1.55)
60	0.03 (0.01)	0.03 (0.02)	31.69 (30.90)	31.53 (24.82)
180	0.01 (0.01)	0.01 (0.01)	92.20 (18.50)	93.33 (15.49)

g/dL (%w/v): grams per decilitre (%weight/volume); SD: standard deviation.

#### Subjective driving performance

Participants self-rated their driving performance as *above average* following the use of methamphetamine alone (mean score = 69.95), and when used in combination with alcohol (mean score 67.60), *average* following placebo (mean score = 49.95) and *below average* following alcohol (mean score = 37.30). There was a significant main effect of treatment of the condition (*F*_(1, 3)_ = 9.05, *p* < 0.001), with better-perceived performance following the use of methamphetamine, both with and without alcohol, relative to both placebo (both *p* < 0.05) and alcohol alone (both *p* < 0.001).

### Driving performance

**Figure 2.**
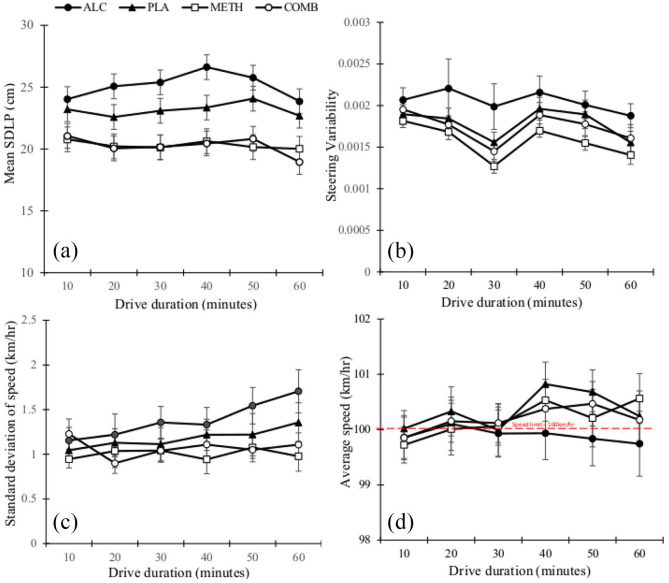
(a–d) Driving performance outcomes are presented as a function of condition and time.

Driving performance parameters are shown in Figure 2, with outcomes presented in Figure 2(a)–(d) as a function of condition and time.

#### Standard deviation of lateral position

There was a significant main effect for condition (*F*_(3, 436)_ = 81.23, *p* < 0.001) and time (*F*_(5, 436)_ = 2.47, *p* *=* 0.032) but not its interaction (*p* = 0.67). SDLP was elevated in the alcohol condition relative to placebo (mean difference = 1.96, *p* < 0.001, 95% confidence interval, CI = 0.98–2.95), methamphetamine (mean difference = 4.81, *p* < 0.001, 95%CI = 3.82–5.79) and combined alcohol and methamphetamine conditions (mean difference = 4.88, *p* < 0.001, 95%CI = 3.89–5.86). Compared to the placebo and alcohol-only conditions, SDLP was significantly reduced following methamphetamine and combined alcohol and methamphetamine (all *p* < 0.05). SDLP was comparable between the methamphetamine and combined alcohol and methamphetamine conditions (*p* > 0.05). A main effect of time was noted for alcohol (*F*_(5, 399)_ = 2.61, *p* *=* 0.02), whereby equivalent SDLP was reported for all conditions at drive start (first 10-min bin, all *p* > 0.05) before divergent and sustained effects were observed between the alcohol relative to the methamphetamine and combined alcohol and methamphetamine condition(s) (all *p* < 0.05) from 20 min until drive conclusion (60 min). At drive conclusion, SDLP was comparable between the alcohol and placebo conditions (*p* > 0.05). No differences were noted between the methamphetamine and combined alcohol and methamphetamine condition at any timepoint (all *p* > 0.05). There was no significant correlation between whole blood concentrations of alcohol or methamphetamine and SDLP in any active condition (*p* *>* 0.05).

#### Steering variability

There was a main effect of condition (*F*_(3, 436)_ = 15.52, *p* *<* 0.001) and time for SV (*F*_(5, 436)_ = 6.63, *p* *<* 0.001), but not its interaction, with greater variability following alcohol use relative to placebo (mean difference = 0.0003, *p* = 0.018, 95%CI = 0.00007–0.0005), methamphetamine (mean difference = 0.0005, *p* < 0.001, 95%CI = 0.0003–0.001) and its combination (mean difference = 0.0003, *p* = 0.03, 95%CI = 0.0001–0.001). SV was largely comparable between conditions over time; however, diverged at 30 min, with increased SV for alcohol, relative to methamphetamine at this timepoint (mean difference = 0.001, *p* = 0.03, 95%CI = 0.0002–0.0012). There was no significant correlation between whole blood concentrations of alcohol or methamphetamine and SV in any active condition (*p* *>* 0.05).

#### Standard deviation of speed

There was a significant main effect of condition for SDS (*F*_(3, 436)_ = 11.44, *p* < 0.001), but not for time or its interaction (both *p* > 0.05). Relative to placebo, SDS increased following alcohol (mean difference = 0.21, *p* = 0.20, 95%CI = 0.22–0.39), but was unchanged following methamphetamine or combined alcohol and methamphetamine use (both *p* > 0.05). SDS increased in the alcohol condition compared to methamphetamine and combined alcohol and methamphetamine condition(s) (mean difference = 0.38, 95%CI = 1.99–0.58, and 0.11, 95%CI = 0.13–0.50), respectively, both *p* < 0.001). There was no significant correlation between whole blood concentrations of alcohol or methamphetamine and SDS in any active condition (*p* *>* 0.05).

#### Average speed (km/h)

There was a significant main effect of condition for average speed (km/h; *F*_(3, 436)_ = 3.05, *p* = 0.03), whereby mean speed (km/h) was reduced in the alcohol condition relative to placebo (mean difference = −0.46, *p* = 0.02, 95%CI = −0.7 to −0.05). There was no effect for time or its interaction (both *p* > 0.05). Mean speed (km) was significantly negatively associated with whole blood concentrations of methamphetamine (*R* = −0.62, *p* *=* 0.01) in the combined condition, with no association found for alcohol.

### Ocular parameters

**Figure 3.**
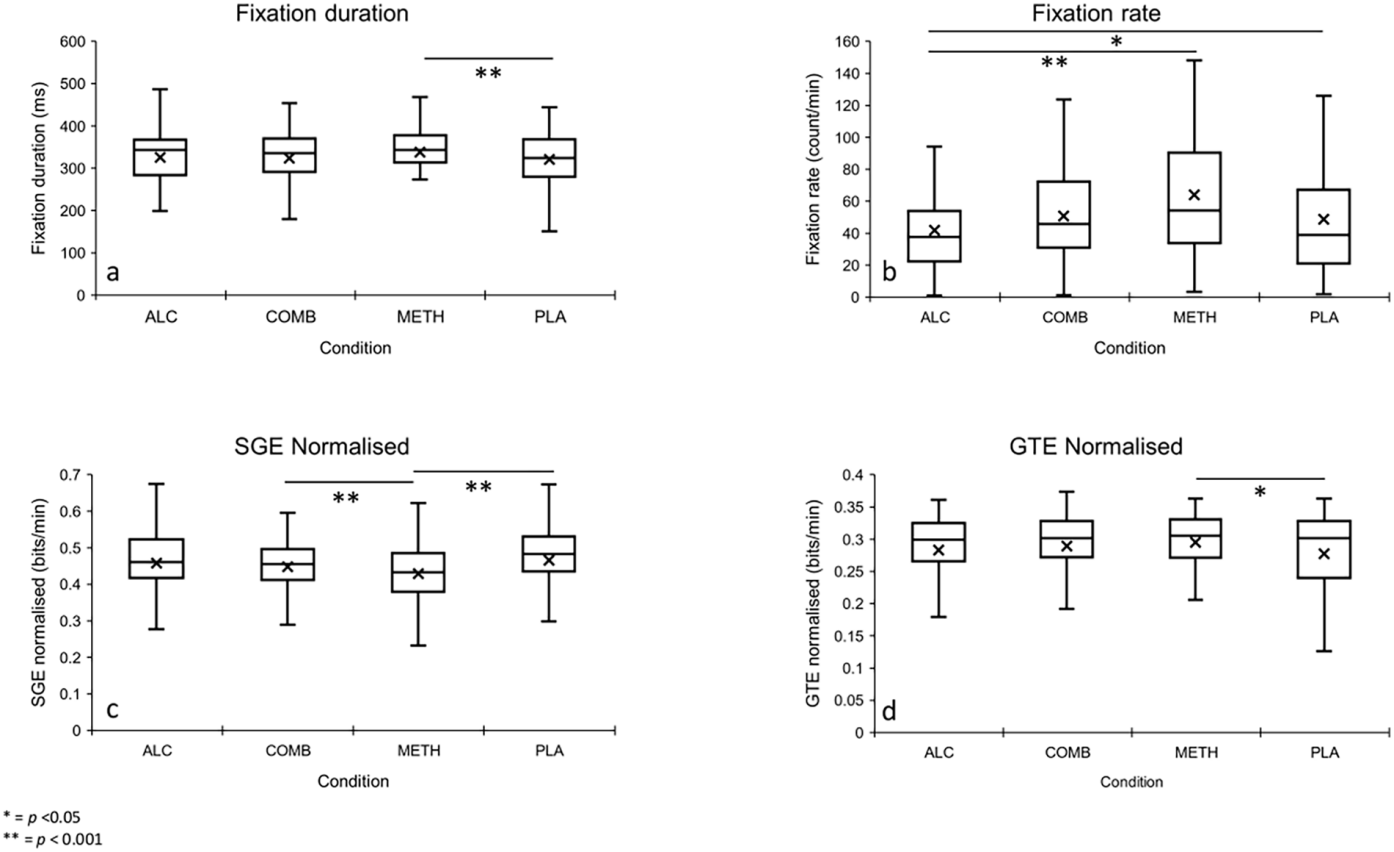
(a–d) Box and whisker plots showing mean, IQR and standard deviations for ocular outcomes by treatment condition. IQR: interquartile range.

Figure 3 illustrates the mean, interquartile range and standard deviations for ocular outcomes by treatment condition. Raw summary data including means and standard deviations (±) for ocular parameters as a function of condition and time are presented in Supplemental Appendix 2.

#### Fixation rate

There was a significant main effect of the condition for fixation rate (*F*_(3, 379)_ = 5.26, *p* = 0.001), but not for time or its interaction (both *p* > 0.05). Methamphetamine significantly increased the number of fixations during driving relative to alcohol alone (mean difference = 20.29, *p* *=* 0.001, 95%CI = 6.08–35.87), but not placebo or combined methamphetamine and alcohol (both *p* > 0.05). Alcohol alone decreased the fixation rate relative to placebo (mean difference = −16.07, *p* = 0.028, 95%CI = −30.95 to −1.16). Increased fixation rate predicted reductions in SDS (Adjusted *R*^2^ = 0.05, *F*_(1, 98)_ = 5.86, β = −0.24, *p* *=* 0.017; SV (Adjusted *R*^2^ = 0.04, *F*_(1, 98)_ = 5.02, β = −0.22, *p* *=* 0.027) and SDLP (Adjusted *R*^2^ = 0.20, *F*_(1, 98)_ = 25.94, β = −0.46, *p* *<* 0.001) in the alcohol only condition.

#### Fixation duration

**Figure 4.**
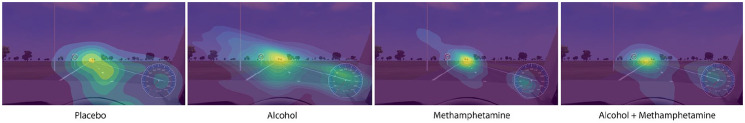
Heatmap overlay of fixations during the simulated driving task for a single participant. Each heatmap is scaled from zero fixations (purple) to the point of maximal fixations (yellow).

A significant main effect of the condition was found for fixation duration (*F*_(3, 379)_ = 2.84, *p* = 0.038). Methamphetamine alone increased fixation duration relative to placebo (mean difference = 19.00, *p* = 0.035, 95%CI = 0.82–37.36), but not the other active treatments. A heatmap (Figure 4) illustrates the location and concentration of fixations for a single participant (participant 18) during the 60-min simulated driving task following each treatment. The heatmap is colour-coded to represent the frequency of fixation with yellow indicating regions with maximal fixation time and purple denoting areas with minimal fixations. Increased fixation duration predicted reduced SDLP (Adjusted *R*^2^ = 0.03, *F*_(1, 99)_ = 4.28, β = −0.20, *p* *=* 0.041) in the alcohol only condition.

#### Stationary gaze entropy

A significant main effect of condition, but not time or its interaction, was found for SGE (*F*_(3, 379)_ = 5.34, *p* = 0.001). Methamphetamine alone significantly reduced SGE during driving, relative to placebo (mean difference = −0.36, *p* < 0.001, 95%CI = −0.062 to −0.12). Whole blood alcohol concentration (g/dL) was moderately positively associated with SGE (Adjusted *R*^2^ = 0.28, *p* = 0.038) in the alcohol-only condition. No other correlations were significant. An increase in SGE predicted increased SDLP (Adjusted *R*^2^ = 0.18, *F*_(1, 98)_ = 22.09, β = 0.43, *p* *<* 0.001), SDS (Adjusted *R*^2^ = 0.16, *F*_(1, 98)_ = 20.37, β = 0.41, *p* *<* 0.001) and SV (Adjusted *R*^2^ = 0.17, *F*_(1, 98)_ = 21.23, β = 0.42, *p* *<* 0.001) in the alcohol-only condition.

#### Gaze transition entropy

There was a significant main effect of condition, but not time or its interaction for GTE (*F*_(3, 379)_ = 4.39, *p* = 0.005). Methamphetamine increased GTE relative to both alcohol (mean difference = 0.17, *p* *=* 0.009, 95%CI = 0.003–0.031) and placebo (mean difference = 0.015, *p* = 0.028, 95%CI = 0.001–0.029), but did not change under the combination of methamphetamine and alcohol condition (*p* *>* 0.05). No other comparisons were found to be significant. Whole blood alcohol concentration (g/dL) was moderately positively associated with GTE (Adjusted *R*^2^ = 0.25, *p* = 0.049) in the alcohol-only condition. No other correlations were significant.

## Discussion

This study is the first to investigate the impact of methamphetamine, alone and in combination with alcohol, on performance and ocular activity during simulated highway driving. Methamphetamine produced a less explorative but more disorganised pattern of gaze during highway driving without negatively affecting performance, which was not changed by the addition of alcohol. Alcohol alone impaired both oculomotor control and driving performance, even when consumed at levels that are well below the legal limit for driving that is stipulated in many jurisdictions. These findings provide new evidence for how these commonly co-consumed yet pharmacologically opposing substances affect visual information processing capabilities during driving and support the need for greater automation of advanced vehicular safety systems that can effectively detect, and index, altered driver state.

Methamphetamine-intoxicated drivers exhibited more frequent and longer visual fixations, reflecting greater cognitive and attentional demand when driving under the influence of this drug compared to alcohol-affected or sober states. Extended periods of visual fixation can impact the initiation and continuity of eye movements that occur between visual points of interest (Shiferaw et al., 2019a), limiting the overall topographic map from which information can be effectively sampled and processed (Arkell et al., 2022b). Supporting this, we show that methamphetamine intoxication reduces SGE while increasing GTE, indicating a less dispersed (more restricted) but more disorganised pattern of visual scanning during highway driving. Functionally, this supports our earlier conceptualisation of methamphetamine-induced dysregulation of oculomotor capabilities, resulting in less efficient scanning of the visual environment and more fixations on the same regions of the visual field (Hayley et al., 2021). As a result, while driving under the influence of this substance, there is less exploratory visual scanning and inefficient visual processing of the environment. As a functional example, a driver who is only looking forward (i.e. narrow field of view) could be moving the eyes between lane markings to maintain position or, their movement patterns could be less structured/intentional moving somewhat randomly although limited within the central region. Psychostimulant intoxication at this same 0.42 mg/kg oral dose has previously been reported to impair signal adherence, braking and car-following distance, reflecting impairment in visual-search tasks during simulated driving (Stough et al., 2012). Protracted amphetamine use is similarly associated with a reduction in visual skills when identifying traffic hazards and an increase in traffic violations due to visual attentional demands, such as running a red light (Tabibi et al., 2021). Considering the broader road safety implications of the current findings, drivers who are acutely intoxicated by methamphetamine and have long-term methamphetamine usage patterns will likely display a similarly reduced capacity to track moving targets (e.g. other moving vehicles or pedestrians; Arkell et al., 2022b), be less able to identify and appropriately react to sudden changes in the road and traffic environment, and thus increase the risk of a collision.

Consistent with existing, but limited human research in this area (Narayan et al., 2021), moderate weighted acute doses of orally ingested methamphetamine inhibited progressive performance deterioration and this was unaffected following the addition of low levels of alcohol. The absence of synergistic and/or additive alcohol-related effects may reflect the low dose explored in this paradigm, or the pervasiveness of the stimulative effects over the sedative effects of alcohol (Hayley et al., 2023). Thus, additional work should be conducted to investigate the impact of higher dosages of alcohol in combination with this psychostimulant. Indeed, our recent review in this area illustrated that any isolated, unidimensional pro-cognitive and behavioural effects of amphetamine appear to be extinguished when alcohol is co-consumed; and, at larger doses, the extent of performance deficits is markedly greater than for either substance alone (Narayan et al., 2021). This appears to be amplified with increasing task complexity, tasks involving higher-order cognitive processes, and multitasking, such as those necessary when driving. As the driving task employed here was a monotonous highway scenario, and the combined alcohol doses were relatively low, it is possible that these task and drug-specific effects were not adequately captured. Additional research should therefore implement a more complex road environment to better define the magnitude of ocular change in tasks of increasing complexity and mental load requirements. Alcohol alone impaired both oculomotor control and driving performance. Even at these modest levels of consumption, the magnitude of change in ocular parameters was found to be associated with systemic alcohol concentrations. Moderate doses of alcohol (0.6 g/kg) have been found to significantly alter both SGE and GTE during a visual-search task, resulting in less explorative gaze distribution and reduced visual scanning efficiency (Shiferaw et al., 2019b). These methods have also been shown to be an efficient way of mapping the ascending and descending phases of alcohol intoxication (Shiferaw et al., 2019b), sleep deprivation (Shiferaw et al., 2018) and the degree to which they mediate and reasonably predict associated impairment in driving performance (Shiferaw et al., 2019c). Given the pharmacologically disparate effects of alcohol and psychostimulants, our data support a differential effect profile, which can be assessed and measured using eye monitoring while driving. Direct evaluation of alterations in eye movement patterns is therefore a promising, non-invasive method for objectively determining visible indicators of intoxication and quantifying the influence of methamphetamine and/or alcohol consumption on driving performance (Stapleton et al., 1986). Extrapolation of such metrics into more complex scenarios during engagement in naturalistic tasks (such as on-track or on-road driving) necessitates a more targeted investigation, especially considering the complex and highly interactive nature of methamphetamine intoxication and task difficulty, which can range from enhancement to deterioration.

This study is strengthened by a randomised, within-subjects controlled crossover design, enabling us to quantify changes in driving performance using a validated simulator with simultaneous eye monitoring capabilities. There are also, however, some notable limitations. Firstly, the use of an acute, low to moderate dose of orally ingested methamphetamine limits the extrapolation of our findings to other, more common (recreational) consumption routes, such as smoking or intravenous injection. Secondly, the use of a single oral alcohol dose, rather than a continuous infusion protocol precludes a more detailed pharmacokinetic investigation. The pharmacokinetic variability produced by the simultaneous (rather than stepped) oral administration of methamphetamine with alcohol may contribute to a misestimation of the magnitude of the observed effect. Therefore, it is strongly recommended that future co-administration protocols apply a more closely controlled administration schedule to accurately capture the anticipated time-to-peak effects. We are also unable to comment on the impact of the findings of either substance under chronic or protracted consumption patterns. Such an experimental intervention is neither ethical nor feasible in laboratory settings, and thus any future work seeking to quantify the magnitude of these effects would benefit from recruiting cohorts of individuals who already self-report heavier recreational consumption of methamphetamine. As the principal outcomes of interest were performance-based effects under the chosen drug conditions, we did not include a baseline control measure. We acknowledge that this may mean that we miss a small amount of data pertaining to individual differences pre-post dosing. While useful for quantifying movement of the eye during driving, the cap-mounted eye tracking system employed here has limited application beyond our highly controlled research environment. Therefore, future research should aim to replicate our findings using static (vehicle-mounted) systems that are less obstructive, allowing for improved monitoring of eye movement behaviour under more naturalistic conditions. The use of a standard highway drive may underestimate the magnitude of ocular changes, and adopting a more complex road environment could help elucidate the true impact of task load and substance-specific effects upon gaze behaviour.

Methamphetamine usage contributes to significant morbidity and mortality on our roads. Despite this, establishing a clear concentration–effect relationship between methamphetamine consumption and traffic crash risk remains challenging. Moreover, current safety countermeasures and enforcement approaches are insufficient in mitigating crash risks for all road users. We show that acute methamphetamine consumption produces a more concentrated, but less efficient exploration of gaze during driving, and even low doses of alcohol produce characteristic alterations to eye movements that can be effectively indexed and monitored by examination of gaze behaviour. Thus, there is an exciting opportunity to explore more proactive approaches in identifying drug-impaired driving by improving systems technologies that monitor and evaluate driver states to prevent intoxicated driving.

### Significance

This is the first randomised controlled clinical trial to examine the acute effects of methamphetamine alone and in combination with alcohol on ocular activity during simulated driving. Technologies that detect and monitor naturalistic gaze behaviours thus have the potential for indexing amphetamine-related driver impairment and inferring driver risk.

## Supplemental Material

sj-docx-1-jop-10.1177_02698811241261024 – Supplemental material for Acute methamphetamine and alcohol usage alters gaze behaviour during driving: A randomised, double-blind, placebo-controlled study Supplemental material, sj-docx-1-jop-10.1177_02698811241261024 for Acute methamphetamine and alcohol usage alters gaze behaviour during driving: A randomised, double-blind, placebo-controlled study by Amie C Hayley, Brook Shiferaw, Blair Aitken, Joanna Rositano and Luke A Downey in Journal of Psychopharmacology

sj-docx-2-jop-10.1177_02698811241261024 – Supplemental material for Acute methamphetamine and alcohol usage alters gaze behaviour during driving: A randomised, double-blind, placebo-controlled study Supplemental material, sj-docx-2-jop-10.1177_02698811241261024 for Acute methamphetamine and alcohol usage alters gaze behaviour during driving: A randomised, double-blind, placebo-controlled study by Amie C Hayley, Brook Shiferaw, Blair Aitken, Joanna Rositano and Luke A Downey in Journal of Psychopharmacology
