# Supplementary material for: Acute methamphetamine and alcohol usage alters gaze behaviour during driving: A randomised, double-blind, placebo-controlled study
Source: J Psychopharmacol. 2024 Jul 28;38(7):636–46. doi: 10.1177/02698811241261024 (PMC11290035; doi:10.1177/02698811241261024)
Supplement: sj-docx-1-jop-10.1177_02698811241261024 – Supplemental material for Acute methamphetamine and alcohol usage alters gaze behaviour during driving: A randomised, double-blind, placebo-controlled study [file sj-docx-1-jop-10.1177_02698811241261024.docx]

Appendix 2. Ocular parameters overall and across time and treatments presented as mean (±SD).

|  |  |  | **Drive time (minutes)** | | | | | |
| --- | --- | --- | --- | --- | --- | --- | --- | --- |
| Treatment | **Outcome** | **Overall** | **10** | **20** | **30** | **40** | **50** | **60** |
| Placebo | SGE (bits/min) | 0.47 (0.10) | 0.44 (0.09) | 0.45 (0.12) | 0.46 (0.12) | 0.49 (0.10) | 0.48 (0.10) | 0.48 (0.08) |
|  | GTE (bits/min) | 0.28 (0.06) | 0.28 (0.06) | 0.27 (0.07) | 0.27 (0.07) | .28 (0.06) | 0.28 (0.07) | 0.29 (0.07) |
|  | Fixation duration (ms) | 320.49 (70.01) | 326.62 (57.05) | 313.07 (71.76) | 306.08 (85.62) | 321.62 (64.61) | 320.74 (73.34) | 335.20 (70.74) |
|  | Fixation rate (count/min) | 56.97 (71.20) | 55.56 (46.60) | 79.57 (120.43) | 75.18 (104.56) | 41.42 (26.88) | 47.60 (35.82) | 42.39 (27.21) |
| Alcohol | SGE (bits/min) | 0.45 (0.10) | 0.45 (0.10) | 0.46 (0.08) | 0.46 (0.09) | 0.45 (0.09) | 0.45 (0.12) | 0.44 (0.15) |
|  | GTE (bits/min) | 0.28 (0.07) | 0.27 (0.04) | 0.27 (0.07) | 0.28 (0.07) | 0.28 (0.06) | 0.28 (0.06) | 0.28 (0.06) |
|  | Fixation duration (ms) | 325.61 (75.78) | 335.64 (67.87) | 313.21 (85.23) | 314.83 (72.77) | 328.48 (63.39) | 333.94 (74.07) | 327.66 (96.52) |
|  | Fixation rate (count/min) | 41.45 (29.21) | 42.68 (32.66) | 42.70 (36.17) | 43.29 (30.10) | 39.71 (28.98) | 39.53 (22.65) | 40.74 (27.027) |
| Methamphetamine | SGE (bits/min) | 0.43 (0.08) | 0.42 (0.09) | 0.44 (0.08) | 0.43 (0.07) | 0.44 (0.08) | 0.42 (0.09) | 0.41 (0.08) |
|  | GTE (bits/min) | 0.29 (0.05) | 0.30 (0.04) | 0.29 (0.05) | 0.30 (0.04) | 0.30 (0.04) | 0.30 (0.05) | 0.29 (0.06) |
|  | Fixation duration (ms) | 338.19 (59.86) | 347.94 (58.07) | 320.49 (63.06) | 336.80 (67.52) | 338.00 (48.78) | 345.26 (59.85) | 340.15 (64.91) |
|  | Fixation rate (count/min) | 64.10 (40.99) | 60.61 (34.12) | 65.73 (61.77) | 61.68 (41.10) | 65.00 (39.84) | 66.68 (32.42) | 64.72 (36.47) |
| Methamphetamine and alcohol | SGE (bits/min) | 0.45 (0.08) | 0.45 (0.08) | 0.45 (0.09) | 0.46 (0.07) | 0.45 (0.09) | 0.45 (0.07) | 0.43 (0.07) |
|  | GTE (bits/min) | 0.29 (0.06) | 0.29 (0.04) | 0.28 (0.07) | 0.29 (0.06) | 0.30 (0.06) | 0.29 (0.06) | 0.28 (0.06) |
|  | Fixation duration (ms) | 323.85 (69.43) | 324.11 (94.40) | 314.38 (76.61) | 324.12 (60.88) | 328.96 (57.50) | 324.70 (66.85) | 326.86 (62.20) |
|  | Fixation rate (count/min) | 50.69 (28.55) | 43.12 (25.21) | 48.54 (29.92) | 48.45 (27.91) | 52.79 (28.40) | 51.07 (28.15) | 60.18 (32.18) |

min = minute; ms = milliseconds; SGE = Stationary gaze entropy; GTE = Gaze transition entropy.
